# Medical Examinations for Life Insurance Companies

**Published:** 1895-03

**Authors:** Jas. N. West

**Affiliations:** Toccoa, Ga.


### MEDICAL EXAMINATIONS FOR LIFE INSURANCE COMPANIES.

By JAS. N. WEST, M.D.,
Toccoa, Ga.

What relation does a physician bear to any life insurance company for which he is medical examiner?

I hold that he bears the same relation to the company that he does to any patient he has charge of, and that the obligations are equally as great, if not greater. Why?

1. Because he has been recommended to the company as an honest, upright, conscientious, painstaking, and intelligent man, capable of filling the office.

2. Because he is paid for his services, and the truth and knowledge which he possesses is expected of him, else he would neither have been appointed nor his services paid for.

3. Because the success of any life insurance company very largely depends upon the ability, integrity, and truthfulness of the medical examiner.

"A physician can with no more impunity hazard his reputation with careless and indifferent examinations, than he can, by the same means, arrive at a false diagnosis or prognosis in a private patient. The consequences of the latter mistakes are well known to the profession, and by reason of the system of interchange of the causes of rejection of applicants by life insurance companies, the former will prove equally as disastrous to the examiner's reputation."

The relation an applicant bears to him is different from that of a patient to his physician. Why?

Because a patient generally exposes and willingly confides every secret of his physical being to his physician, so that he may more fully understand and intelligently treat his case. This is not the case with an applicant for insurance. He seeks to avoid making known his complaints and tries to suppress all knowledge of any malady he may have been or is suffering from at present; he can better do this with an examiner who is not his regular physician. What is the duty of the examiner who is his regular physician?

The best policy is to tell the truth under all circumstances, and never allow personal friendship as an agent to influence you one particle towards making up your approval or disapproval of an applicant. Never make a compromise with honor and truth.

You should make your examinations in your office and with no one present but yourself and the applicant. Why? Because you can better devote your mind to your work, and under all circumstances avoid the annoying hanging-round agent, who is always anxious to push applicants through; and last and most important, because applicant will give you a more complete history of past troubles and his present condition if you two are alone.

His family and personal history are of great importance to you in making up your judgment, and equally of as much importance to the medical director, as to whether he should be accepted or rejected. Being perfectly familiar from youth up with the applicant's life and habits should not at all influence you as to the thoroughness of the examination at the stated time. You are paid for a genuine and thorough examination, and let there be no guesswork about it. Let his personal appearance affect you not at all.

When your examination goes before the medical director, he supposes you have used every possible means of coming to an accurate and truthful statement. You should go over the examination twice before you dismiss the applicant and see that the answers are made true and plain. Don't allow yourself to use the words "childbirth," "general debility," "change of life," "liver complaint," "intemperance," "don't know," etc.; and if the cause of death of some relative is not known and cannot be ascertained, use the words "unknown to me."

They sound better and fill all demands. You must endeavor in filling out the blanks furnished by the company to give every answer full and complete, so that the knowledge furnished by you will be sufficient for the medical director to have the necessary information on the application.

Never leave a blank unanswered or run your pen through it; it makes an ugly and untidy appearance to say the least of it.

I notice the companies attach more importance to intemperance in the use of intoxicants now than heretofore. So, unless you want your examination blank sent back for "completion," don't give as an answer, "takes a drink occasionally." State the amount daily, weekly, as the case may be. If he is an abstainer have him state over his signature how long he has been one. Invariably date your examination the day it is made.

When an answer to a question on applicant's statement has to be erased, write the correct one in a neat hand over the other, being careful always to have the applicant write his initials by the erasure, for he alone has the right to make any changes in his statement after the signatures have been written.

In case you are compelled to make an erasure in your report do likewise, signing your own initials.

If applicant is unknown to you, refuse to make the examination unless a satisfactory introduction by one who knows him to be the one named in the application.

It is better in making answers to questions to say "Yes" or "No" when it will meet all requirements. Explain all technical names for diseases to the applicant, such as "phthisis," "hemoptysis," "fistula," etc.

Never neglect to weigh the applicant yourself. If any characteristic in family, state plainly. Always measure his chest with top shirt off, as well as making your physical examinations of lungs and heart.

In stating colic be particular to state kind, whether renal, hepatic, or intestinal. Always state frequency of attacks. See yourself that there are always signs of successful vaccination. Inquire into past history for inflammatory diseases, such as rheumatism, how many attacks, how far apart, and parts affected. If he has had a limb amputated, state which one and where, and whether from disease or injury; if disease, state its nature. State whether he is able to wear artificial limb or not. Any history of hemoptysis should be fully examined. Otorrhea should be fully explained and the nature of discharge given. In stating duration of illness don't fail to state length of time affected and not the length of time in bed.

In making examinations of urine see that specimen is voided in your presence and into a clean bottle. Make your examination at once, as the urine goes through chemical changes. You should never fail to make thorough examinations of the urinary organs, for catarrh of bladder, stricture, enlargement of prostate, calculus, gonorrhea, excessive micturition, etc. I use nitric acid and heat test for albumin. Test for sugar, I use Boettchers, Glycerine Cupric, and Haines tests.

